# Prevalence of antiphospholipid syndrome among women with recurrent pregnancy loss: a cohort study

**DOI:** 10.61622/rbgo/2025rbgo23

**Published:** 2025-04-30

**Authors:** Elaine Cristina Fontes de Oliveira, Daniel Dias Ribeiro, Janaína Campos Senra, Fernando Marcos dos Reis

**Affiliations:** 1 Universidade Federal de Minas Gerais Hospital das Clínicas Division of Human Reproduction Belo Horizonte Minas Gerais Brazil Division of Human Reproduction, Hospital das Clínicas, Universidade Federal de Minas Gerais, Belo Horizonte, Minas Gerais, Brazil.; 2 Universidade Federal de Minas Gerais Hospital das Clínicas Departament of Hematology Belo Horizonte Minas Gerais Brazil Departament of Hematology, Hospital das Clínicas, Universidade Federal de Minas Gerais, Belo Horizonte, Minas Gerais, Brazil.

**Keywords:** Abortion habitual, Abortion spontaneous, Antibodies, Anticardiolipin, Antiphospholipid syndrome, Prevalence, Thrombophilia

## Abstract

**Objective:**

This study aimed to evaluate the prevalence of antiphospholipid syndrome (APS) among women experiencing recurrent pregnancy loss (RPL).

**Methods:**

A cross-sectional was conducted, reviewing the medical records of 134 women with a history of two or more miscarriages, treated between January 2014 and May 2024 at a tertiary university center in Belo Horizonte, Brazil. APS screening was performed by assessing anticardiolipin (IgG and IgM), lupus anticoagulant, and anti-β2-glycoprotein-1 (IgG and IgM) antibodies, based on Sapporo criteria. All tests were performed during non-pregnant periods and at least 12 weeks after the last miscarriage.

**Results:**

The study included 134 women with a mean age of 33.8 ± 5.7 years. The number of prior miscarriages ranged from 2 to 11 per couple. Among the patients who presented the lupus anticoagulant, only two (1.49%) tested positive in two samples, as per revised Sapporo criteria. Considering IgG and IgM anticardiolipin antibodies, four patients (2.98%) tested positive in two samples according to old Sapporo criteria, with one patient having a positive IgG test in two samples, two having positive IgM in two samples and a single patient having both positive tests. None of the 56 patients tested positive for anti-β2-glycoprotein-1 antibodies in two samples.

**Conclusion:**

The prevalence of antiphospholipid antibodies, in line with revised Sapporo criteria, is low among Brazilian women with recurrent pregnancy loss, consistent with recent studies in literature. Ensuring the appropriateness of diagnostic criteria is crucial to avoid unnecessary treatment with platelet anticoagulants and heparin in this population.

## Introduction

Recurrent pregnancy loss (RPL) is traditionally defined as the loss of three consecutive pregnancies before 20 weeks of gestation.^(1)^ However, there are variations of this definition in literature, considering the number, the timing of losses, and the type of pregnancy evaluated.^(2-4)^ Both the European Society of Human Reproduction and Embryology (ESHRE) and the American Society for Reproductive Medicine (ASRM) consider only clinical pregnancies (those diagnosed by ultrasound or anatomopathological examination) for diagnosis, excluding molar and ectopic pregnancies.^(2,3)^ Conversely, the Royal College of Obstetricians and Gynaecologists (RCOG) includes biochemical pregnancies as well.^(4)^

The prevalence of RPL varies depending on how early the pregnancy is recognized and the criteria used for its definition.^(5)^ It affects approximately 3% of couples attempting to conceive when considering at least two losses, and about 1% when considering three or more losses.^(1)^ As the prevalence of alterations in the investigation for RPL does not vary in couples with two or three losses,^(6)^ it is currently recommended to search for causal factors after two miscarriages.

Recurrent pregnancy loss is part of the gestational complications of the first and second trimesters. Many losses (40%) occur before they are clinically recognized. Considering recognized pregnancies, about 15% result in miscarriage.^(1,7)^ Unlike sporadic losses, RPL is considered pathological, requires investigation and sometimes therapeutic interventions, as well as emotional support and monitoring during future pregnancies.^(5)^

RPL has multiple etiologies, with various contributing factors. Among the well-established risk factors for RPL, antiphospholipid syndrome (APS) stands out.^(8-11)^ Various studies report a strong association between specific antiphospholipid antibodies (aPL) and APS with RPL.^(12)^

APS is an autoimmune condition characterized by the presence of antibodies, vascular thrombosis, and gestational morbidity. APS occurs alone or in association with other autoimmune diseases, particularly systemic lupus erythematosus (SLE).^(8,13-16)^ According to the Sydney classification criteria ("revised Sapporo criteria"),^(16)^ the clinical manifestations should be associated with the persistent positivity for aPL.^(8,16,17)^ In early losses, the involved mechanism appears to be related to the formation of an ineffective buffer system, secondary to failure in initial trophoblastic invasion, which increases exposure to stressors or direct trophoblast injury by antibodies.^(8,17,18)^

The ESHRE recommends screening for APS by performing assays for IgG and IgM anticardiolipin and lupus anticoagulant antibodies. The screening for anti-β2-glycoprotein-1 can be considered after two gestational losses.^(2)^ The ASRM recommends carrying out the three tests.^(3)^ On its turn, the RCOG recommends measuring anticardiolipin and lupus anticoagulant antibodies on two different occasions, within a 12-week interval.^(4)^

Previous studies have documented APS in 15-20% of women with RPL.^(6,9-12)^ However, the redefinition of diagnosis by the revised Sapporo criteria, which require higher antibody levels (anticardiolipin antibodies ≥ 40 U/mL and dRVVT ratio ≥ 1.2 for lupus anticoagulant) and their reassessment after a 12-week interval resulted in prevalence rates of APS in RPL women similar to the general population.^(19,20)^ The prevalence of APS varies among different populations^(8,12)^ and its reassessment using current diagnostic criteria is essential to guide screening protocols among women with RPL, thereby avoiding overdiagnosis and overtreatment. Thus, the aim of this study is to evaluate the prevalence of APS in Brazilian women with RPL.

## Methods

This cross-sectional study reviewed the medical records of women assisted on RPL Clinic at the University Hospital of Belo Horizonte, Brazil, between January 2014 and May 2024. These women were referred from several other services and hospitals in the region. They were included consecutively, with no prior selection before the initial consultation.

Participants included were women of any age, race, or socioeconomic status diagnosed with RPL. RPL was defined as a history of at least two clinical pregnancies (i.e., confirmed by ultrasound and/or clinical examination) spontaneously interrupted before 20 weeks of gestation. Patients who missed IgM and IgG anticardiolipin and lupus anticoagulant measurements were excluded.

All patients underwent routine screening tests including couples’ karyotype, APS screening, serum thyroid stimulating hormone (TSH), serum prolactin (if clinical suspicion of hyperprolactinemia), transvaginal ultrasound, and hysteroscopy. APS screening was performed through assays for anticardiolipin (IgG and IgM), lupus anticoagulant, and anti-β2-glycoprotein-1 (IgG and IgM) antibodies. These tests were conducted during non-pregnant periods and at least 12 weeks after the last miscarriage. Anticardiolipin and anti-β2-glycoprotein-1 antibodies were analyzed using a fluoroimmunoassay/ELISA. Lupus anticoagulant was analyzed using PTTa and diluted Russell's viper venom time (DRVVT) or Silica Clotting Assay and DRVVT. The laboratory criteria for APS included the detection of lupus anticoagulant, IgG/IgM anticardiolipin antibodies with titers >40 GPL or MPL or >99th percentile or IgG/IgM anti-β2glycoprotein-1 antibodies with titers >99th percentile. Lupus anticoagulant positive serum test result was considered abnormal according to our in-house reference. A second assay was performed after a 12-week interval for diagnosis confirmation.

Patients with positive results for any of these tests were referred to the Hematology Clinic for preconception counseling, including the use of aspirin periconceptionally and of heparin during pregnancy.

Continuous data were reported as mean ± SD and range (minimum– maximum). The categorical variables were described as percentages. A *post-hoc* test indicated that by surveying 134 participants the margin of error to estimate a population proportion around 3% with 95% confidence level is of ± 2.9%.

Approval for this project was obtained from the Research Ethics Committee of Universidade Federal de Minas Gerais 5.464.119 (*Certificado de Apresentação de Apreciação Ética:* 58024219.3.0000.5149).

## Results

This study included a total of 134 couples with a history of 2 or more recurrent abortions. The mean age of women was 33.8 ± 5.7 years (range: 20 – 46 years), whereas the mean age of male partners was 35.9 ± 6.4 years (range: 21 – 56 years). The number of previous abortions varied from 2 to 11 abortions per couple with a mean of 3.1 ± 1.5. The clinical characteristics of participants are shown in table 1.

**Table 1:** Clinical characteristics of the study participants

Characteristics		Total
Female age (range) (n=134)		33.8 ± 5.7
Male age (range) (n=112)		35.9 ± 6.4
Number of miscarriages		
	2	62
	3	39
	4	16
	5	10
	6	2
	7	3
	9	1
	11	1
Time of pregnancy loss		
	Only first trimester	99
	Only second trimester	1
	First and second trimester	21
	First and third trimester	11
	First, second and third trimester	2

As shown in table 2, of the 134 patients tested for the lupus anticoagulant antibody, only two (1.49%) tested positive in two samples according to the revised Sapporo criteria. All these patients were also tested for IgG and IgM anticardiolipin antibodies. Of these, only four (2.98%) had positive tests in two samples according to the old Sapporo criteria, with one patient having a positive IgG test in two samples, two having positive IgM in two samples and a single patient having both positive tests. It is important to highlight that no patient had antibody levels >40 GPL or MPL. Of the 56 patients tested for anti-β2-glycoprotein-1 antibodies, none (0%) presented a positive test in two samples.

**Table 2:** Prevalence of positivity for antiphospholipid antibodies in patients with Recurrent Pregnancy Loss in a Tertiary Center in Brazil, according to Sapporo criteria

Antiphospholipid antibody	Number of patients tested	Negative test in first sample	Positive test in first sample	Positive test in two samples
Anticardiolipin antibodies	134	128	6*	4*
Lupus anticoagulant	134	131	3	2
Anti-β2-glycoprotein-1	56	54	2*	0*

* IgM or IgG positive test

## Discussion

The frequency of positivity for lupus anticoagulant and anticardiolipin antibodies in women with RPL varies widely in the literature, being reported between 8-42% of cases in different studies.^(3)^ However, older studies described a high prevalence of APS in women with RPL. These studies may present several limitations, such as: lack of distinction between losses before and after 10 weeks; incomplete assessment of other causes of RPL; retrospective design; patients selected prior to the revised Sapporo criteria for APS; inclusion of minimally positive aPL; lack of confirmatory test and few clinical studies testing for anti-β2-glycoprotein-1.^(17,21)^

On the other hand, two recent studies demonstrated a prevalence of aPL similar to the general population. A multicenter retrospective case-control study comparing 820 patients with RPL with a control group of healthy women screened for thrombophilias found the same prevalence of aPL in both groups.^(19)^ In a retrospective cohort study of 1,155 women with a history of at least 3 miscarriages, only 1% of the participants tested positive for acquired thrombophilia, being 0.5% with a persistent positive test for lupus anticoagulant and 0.5% with persistent anticardiolipin antibodies ≥ 40 U/ml.^(20)^ Thus, the prevalence found in our study for anticardiolipin and lupus anticoagulant antibodies was similar to that found in current studies.^(19,20)^

The consensus classification criteria for APS, known as the Sapporo criteria, were initially proposed in 1998 during a post-conference workshop following the Eighth International Symposium on Antiphospholipid Antibodies in Sapporo, Japan. It was subsequently updated at the Eleventh International Congress on Antiphospholipid Antibodies in Sydney in 2006, resulting in the "revised Sapporo criteria." These revisions included the addition of anti-β2-glycoprotein-1 IgM or IgG to the diagnostic criteria, extending the minimum confirmation period from 6 to 12 weeks, and establishing minimum values for anticardiolipin antibodies and anti-β2-glycoprotein-1 antibodies. The initial obstetric criteria remained unchanged. For anticardiolipin antibodies (IgM or IgG) to meet the criteria, they should exceed 40 IgM or IgG antiphospholipid units, or anti-β2-glycoprotein-1 antibodies (IgM or IgG) should be at levels exceeding the 99th percentile, as measured by enzyme-linked immunosorbent assay (ELISA). Given that antiphospholipid antibodies (aPL) may transiently appear in conditions such as viral syndrome or pregnancy, it was recommended to perform these assays on two separate occasions, with at least twelve weeks between tests, to minimize the risk of diagnosing APS based on transient antibodies.^(16,17)^

The aPL are usually measured at a short interval (6 weeks) after the clinical event, followed by a confirmatory test after 12 weeks.^(16-22)^ Since there may be fluctuating levels of these antibodies during pregnancy, which can result in a negative test after birth or abortion,^(17)^ the ideal strategy would be to wait a longer period to measure these antibodies. Although there is no optimal time for collecting those tests, one study found that lupus anticoagulant antibodies return to baseline three months after birth.^(23)^ In our department, the aPL test is carried out 12 weeks after birth or abortion, with the confirmatory test carried out 12 weeks later. Since all patients are asked to investigate other causal factors in RPL, this 12-week interval does not seem to increase the couple's anxiety about an etiological diagnosis. Patients are advised not to become pregnant while undergoing the entire RPL workup.

One limitation of our study was that not all patients were able to perform the anti-β2-glycoprotein-1 measurement, since our hospital did not offer it as a standardized test. So, the prevalence of anti-β2-glycoprotein-1 in our population could not be correctly estimated.

It is well-established that the presence of lupus anticoagulant and a history of venous thromboembolism are key predictors of subsequent adverse pregnancy outcomes.^(24,25)^ However, studies have shown that the solely positivity for IgM or IgG anticardiolipin, and anti-β2-glycoprotein-1 antibodies does not reliably predict adverse pregnancy outcomes.^(24)^ Given the ongoing debate surrounding the role of these antibodies and the unclear clinical significance of low antibody levels, it's crucial to adhere to standardized APS diagnoses, utilizing the revised Sapporo criteria. This standardization is essential for accurately evaluate the treatment efficacy of patients with APS using acetylsalicylic acid and heparin. Further studies should address this topic.

Furthermore, in 2023, an international multidisciplinary initiative introduced an additive, weighted system to evaluate the relative probability of APS for research purposes: the 2023 ACR/EULAR (American College of Rheumatology/European League Against Rheumatism) APS classification criteria. The criteria aimed to include individuals exhibiting key features of APS to form homogeneous cohorts, ensuring comparability across clinical studies and trials. The 2023 ACR/EULAR APS classification criteria included an entry criterion of at least one positive aPL test within 3 years of identification of an aPL-associated clinical criterion, followed by additive weighted criteria (score range 1–7 points each) clustered into 6 clinical domains and 2 laboratory domains (lupus anticoagulant functional coagulation assays, and ELISA for IgG/IgM anticardiolipin and/or IgG/IgM anti–β2-glycoprotein-1 antibodies). Individuals were classified as having APS for research if they accumulate at least 3 points from clinical domains and at least 3 points from laboratory domains. The isolated presence of 3 or more consecutive pre-fetal losses (<10 weeks) and/or early fetal death (10 weeks 0 days – 15 weeks 6 days) presents a weight of one for the clinical criterion of APS. Single (one-time) LAC positivity may be relevant (1 point) when repeat testing is unavaiable (5 points if positive LAC persistent). Both the levels of anticardiolipin and anti-β2-glycoprotein-1 antibodies measured, and the association between them, provide different scores for the laboratory criteria for APS (ranging from 1 to 7 points).^(26)^ So, this new classification system will have important impacts on the prevalence of APS in women with RPL and the consequent evaluation of the effectiveness of usual treatments.

## Conclusion

In conclusion, the prevalence of antiphospholipid antibodies (1.49%), in line with revised Sapporo criteria, is low among Brazilian women with recurrent pregnancy loss, consistent with recent international studies. Ensuring the appropriateness of diagnostic criteria is crucial to avoid unnecessary treatment with platelet anticoagulants and heparin in this population.
